# Stratified layer analysis reveals intrinsic leptin stimulates cryptal mesenchymal cells for controlling mucosal inflammation

**DOI:** 10.1038/s41598-020-75186-3

**Published:** 2020-10-27

**Authors:** Seiichi Matsumura, Yosuke Kurashima, Sayuri Murasaki, Masako Morimoto, Fujimi Arai, Yukari Saito, Nana Katayama, Dayoung Kim, Yutaka Inagaki, Takahiro Kudo, Peter B. Ernst, Toshiaki Shimizu, Hiroshi Kiyono

**Affiliations:** 1 Department of Innovative Medicine, Graduate School of Medicine, Chiba University, 1-8-1 Inohana, Chuo-ku, Chiba-shi, Chiba, 260-8670 Japan; 2 Department of Mucosal Immunology, The University of Tokyo Distinguished Professor Unit, The Institute of Medical Science, The University of Tokyo, Tokyo, 108-8639 Japan; 3 Department of Pediatrics, Juntendo University Faculty of Medicine, 2-1-1 Hongo, Bunkyo-ku, Tokyo, 113-8421 Japan; 4 International Research and Development Center for Mucosal Vaccines, The Institute of Medical Science, The University of Tokyo, Tokyo, 108-8639 Japan; 5 Division of Gastroenterology, Department of Medicine, CU-UCSD Center for Mucosal Immunology, Allergy and Vaccines (CU-UCSD cMAV), University of California, San Diego, CA 92093-0956 USA; 6 Center for Matrix Biology and Medicine, Graduate School of Medicine, Tokai University, Kanagawa, Japan; 7 Division of Comparative Pathology and Medicine, Department of Pathology, University of California San Diego, San Diego, CA 92093-0956 USA; 8 Center for Veterinary Sciences and Comparative Medicine, University of California, San Diego, CA 92093-0956 USA

**Keywords:** Gastrointestinal diseases, Gastrointestinal hormones, Gastrointestinal system, Cell biology, Immunology, Gastroenterology, Anatomy, Cells, Gastrointestinal system

## Abstract

Mesenchymal cells in the crypt play indispensable roles in the maintenance of intestinal epithelial homeostasis through their contribution to the preservation of stem cells. However, the acquisition properties of the production of stem cell niche factors by the mesenchymal cells have not been well elucidated, due to technical limitations regarding the isolation and subsequent molecular and cellular analyses of cryptal mesenchymal cells. To evaluate the function of mesenchymal cells located at the large intestinal crypt, we established a novel method through which cells are harvested according to the histologic layers of mouse colon, and we compared cellular properties between microenvironmental niches, the luminal mucosa and crypts. The gene expression pattern in the cryptal mesenchymal cells showed that receptors of the hormone/cytokine leptin were highly expressed, and we found a decrease in Wnt2b expression under conditions of leptin receptor deficiency, which also induced a delay in cryptal epithelial proliferation. Our novel stratified layer isolation strategies thus revealed new microenvironmental characteristics of colonic mesenchymal cells, including the intrinsic involvement of leptin in the control of mucosal homeostasis.

## Introduction

The intestinal mucosa plays critical physical roles in the ingestion and absorption of necessary nutrients; at the same time, it is exposed to diverse potentially adverse agents, including infectious pathogens and other harmful substances such as allergens and toxins^1,2^. Therefore, intestinal mucosa is equipped with physical (e,g., tight junctions), chemical (e.g., antimicrobial peptides), and immunological (e.g., secretary IgA) barrier systems, with the mucosal epithelial regeneration process executing well-organized and prompt responses to tissue damage or inflammation^1^. Through accumulated past studies, the unique features and dynamics of the mucosal immune system have been well characterized, and immune–epithelial crosstalk for the generation of functional epithelial barriers has been well evaluated^1^. Furthermore, recent evidence has revealed the indispensable roles of mesenchymal cells (e.g., fibroblasts, myofibroblasts, and telocytes) in epithelial and cryptal regeneration^3,4^. However, the molecular and cellular mechanisms underlying the regulation of intestinal mesenchymal cells are relatively unknown, and an understanding of these mechanisms is essential to revealing the mucosal regeneration process and disclosing fundamental therapeutic strategies against intestinal inflammation (e.g., inflammatory bowel diseases, IBD)^1,5,6^.

Accumulated evidence had indicated the importance of the mesenchymal network-based stem cell microenvironment for the creation and maintenance of intestinal homeostasis^6^. Epithelial regeneration is achieved through the proliferation of LGR-5^+^ stem cells in the epithelial cryptal base^7,8^, and the maintenance and differentiation of those stem cells are tightly regulated by the mesenchymal cells around those stem cells^8–10^. Various subpopulations of mesenchymal cells (e.g., intra-sub-epithelial myofibroblasts, GLI family zinc finger 1^+^ [Gli1^+^] fibroblasts, and telocytes) provide niche factors involved in the Wnt/β-catenin pathway, including WNTs, Gremlin (GREM), and Bone Morphogenetic Proteins (BMP)s, that are essential for intestinal renewal and stem cell maintenance^11,12^. However, the mechanisms how the cryptal mesenchymal cells acquire the ability to maintain the stem cell niche are not well elucidated due to the limited methods for isolating cryptal mesenchymal cells for molecular and cellular analyses. To evaluate the precise function of mesenchymal cells located at the large intestinal crypt, reliable isolation methods of cryptal cells based on the histological layers of the colon are required.

The alimentary canal is anatomically and histologically composed of four broad layers: the mucosa, submucosa, muscularis externa (MEx), and serosa^13^. The mucosal layer is subdivided further into three layers: epithelium, lamina propria, and muscularis mucosae (MM)^13^. The compositions of these layers are similar throughout the digestive tract, with partial specialization among mucosal tissues^13^. Importantly, in gut mucosa, mesenchymal cells or stroma cells exist in each layer of the intestinal compartments; therefore, a new technique that facilitates stratified isolation is needed for the study of cryptal mesenchymal cells^6^.

In this study, we sought to uncover the anatomical and histological characteristics of the mesenchymal cells in colonic cryptal microenvironments by using a newly developed intestinal layer isolation method with experimental verification. The subsequent molecular and cellular analysis newly identified several receptors highly expressed in cryptal mesenchymal cells, including receptors for leptin, which is a homeostatic hormone/cytokine derived from adipose tissues. Here we present our novel gut mucosal layer isolation method for the characterization of the mesenchymal cell population in murine colon. Using this strategy, we discovered important roles of intrinsic leptin and its involvement in intestinal mucosal regeneration.

## Results

### Stratified isolation of mouse colon for cryptal cell analysis

That unique mesenchymal populations support cryptal niches in the small intestine and colon is widely accepted^14^. In addition, the roles of mesenchymal cells differ among their histological and microenvironmental locations, such as villus tips and crypt^8,15^. To evaluate the characteristics and function of crypt mesenchymal cells in colon as a first step in uncovering mesenchymal cell-mediated mucosal homeostatic pathways, we developed a stratified isolation method for murine colon that achieved optimal separation of the lumen and crypt parts of the colonic mucosa. To this end, murine colon tissues were isolated according to the 6 histologic components: epithelium, upper mucosa with lamina propria, crypt, MM, submucosa, and MEx^13^.

**Figure 1:**
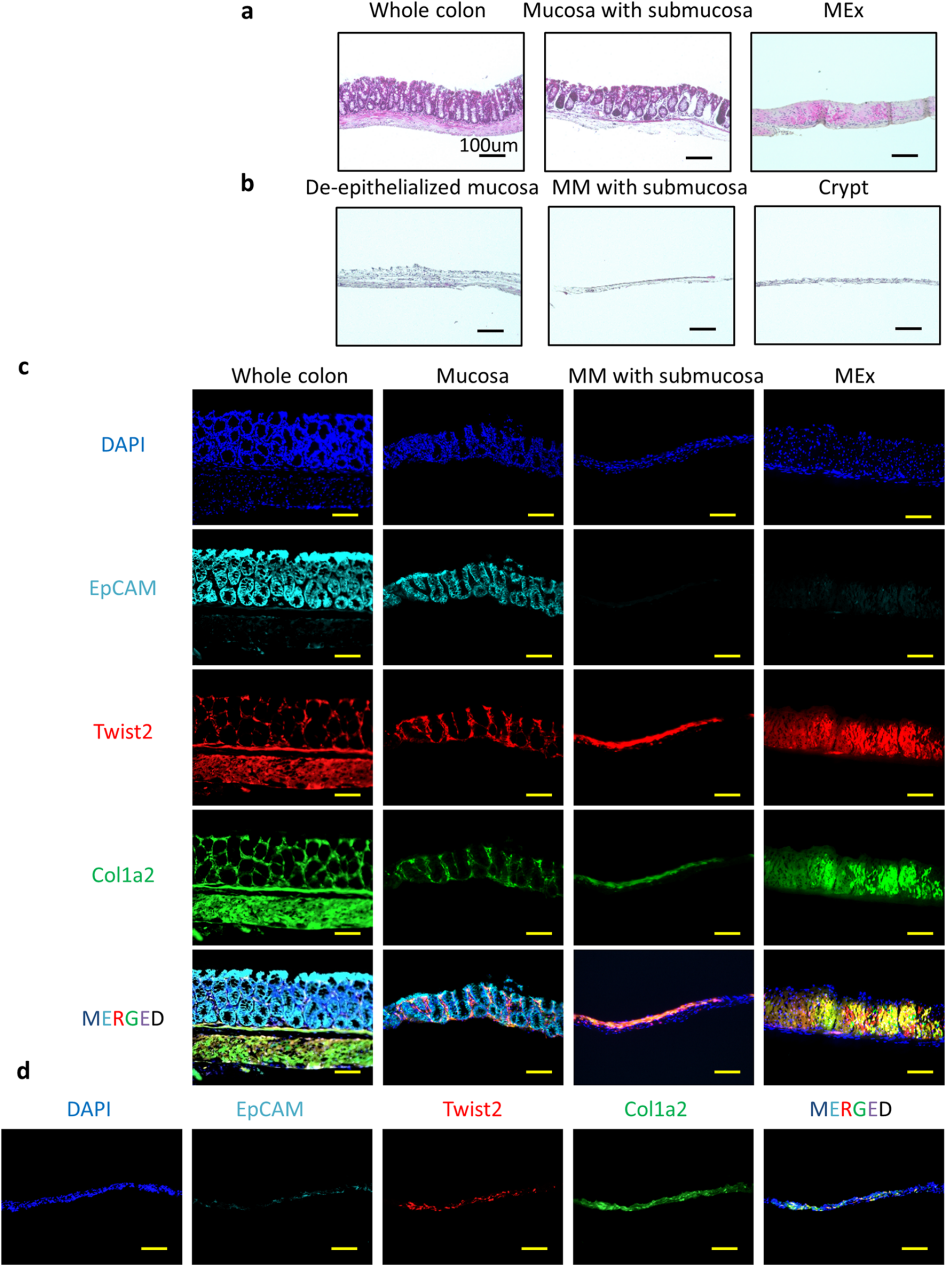
Stratified isolation of mouse colon for microenvironmental cellular analysis. (**a**) Representative images of hematoxylin and eosin (H and E) staining of whole colon, peeled layers containing mucosa and submucosa, and muscularis externa (MEx) are shown. (**b**) Representative images of H and E staining of de-epithelialized mucosa, muscularis mucosae (MM) with submucosa, and crypt layer are shown. Bar, 100 μm. (**c**) Representative images of whole colon, mucosa, MM with submucosa, and MEx from Twist2-Cre tdTomato × Col1a2 GFP mice and stained by using EpCAM are shown. (**d**) Crypt region of Twist2-Cre tdTomato × Col1a2 GFP mice is shown. Bar, 100 μm. Images representative of at least three independent experiments are shown.

First, we separated the MEx from the mucosa and submucosa by using microtweezers (Fig. 1a, Supplemental Fig. 1, and Supplemental Video); these tissues were partly de-epithelialized through incubation in cell dissociation solution (Fig. 1b). Under a stereo-microscope, we then used a scraper and microtweezers to sub-divide the upper mucosal and cryptal compartments with the MM and submucosal compartments (Fig. 1b, Supplemental Fig. 1, and Supplemental Video). Finally, we isolated the cryptal regions from the MM and submucosal compartment (Fig. 1b, Supplemental Fig. 1, and Supplemental Video). Staining with hematoxylin and eosin confirmed the isolation of the various layers and revealed that, through the separation procedure, the various layers of the colonic mucosa had been anatomically and histologically correctly dissected (Fig. 1a,b).

To further validate the experimental procedure, we applied the isolation protocols to tissues from Twist2-Cre-td Tomato–type I Collagen (Col1a2)–EGFP mice which targeted two mesenchymal marker genes (Twist2 and Col1a2)^5^. In these mice, mesenchymal cell populations were stained red in color, and collagen-producing cells in the colonic tissues were green; epithelial cells were stained by the marker EpCAM (Fig. 1c). Whole-colon tissues were separated into 3 parts (mucosa, MM with submucosa, and MEx) (Fig. 1c). Twist2^+^ Col1a2^+^ (yellow-colored) cell populations occurred in the mucosal, MM, and MEx compartments of the mouse colon (Fig. 1c and Supplemental Fig. 2). After the upper mucosa was removed, Twist2^+^ Col1a2^+^ cell populations remained in the isolated cryptal region (Fig. 1d, Supplemental Fig. 2). These histological results confirmed the validity of the stratified isolation method for cells from the murine colonic mucosa.

### Stratified isolation for spatial and phenotypic mesenchymal cellular profiling in the colon microenvironment

**Figure 2:**
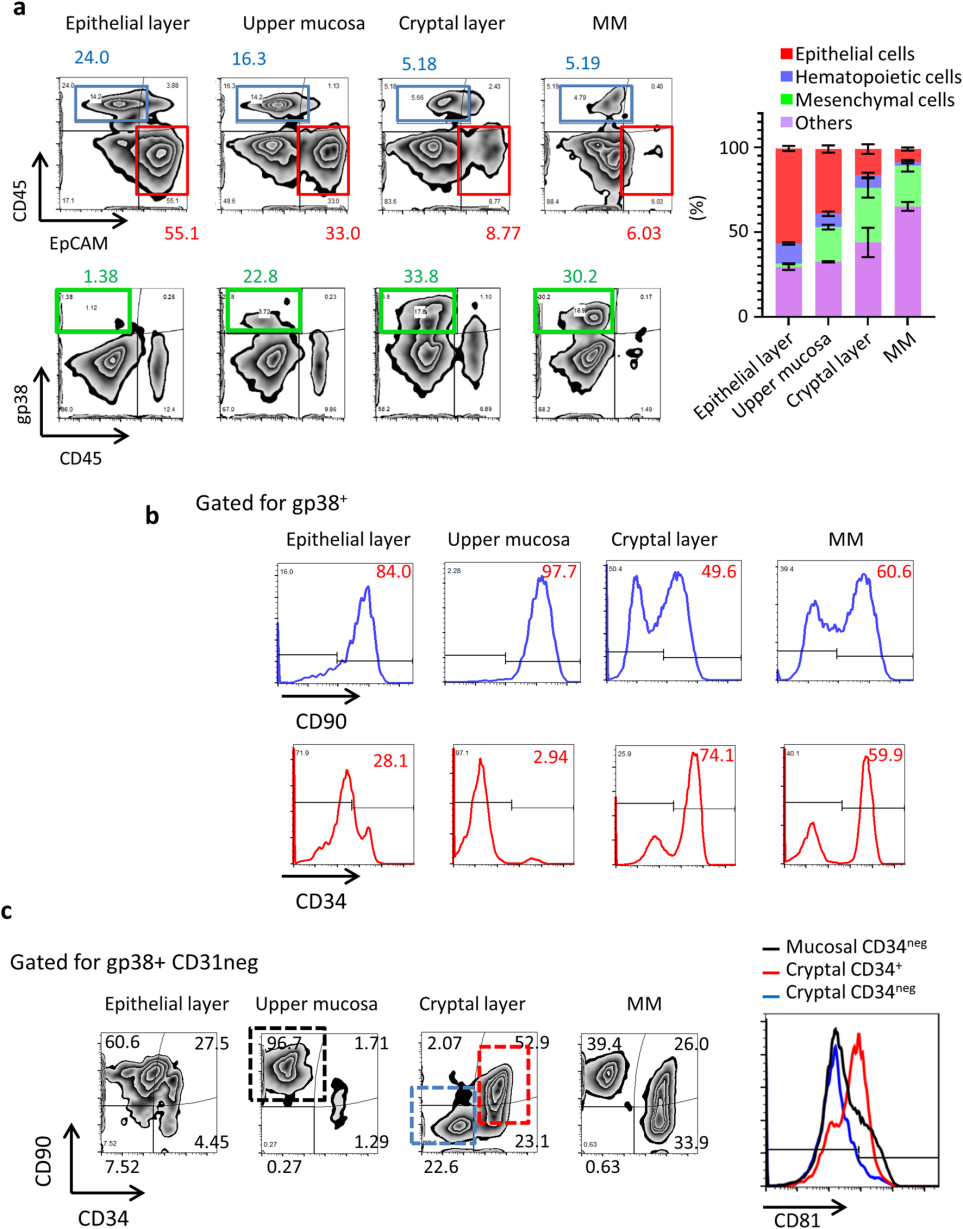
Cellular profiling of stratified isolated cell populations. (**a**) Flow cytometry of stratified isolated cells stained with CD45, EpCAM, and gp38. The number indicates percentages of gated populations. Right data indicates the percentages of each cellular component in the colon microenvironment. Data are shown as means ± SEM. (**b**) Flow cytometry of cells in each layer is shown. The gp38^+^ cells in the negative-gated areas for EpCAM, TER119, and CD31 are shown. The number indicates the size (percentage) of the gated population. CD90 and CD34 expression of gated gp38^+^ cells are shown. (**c**) gp38^+^ cells were stained with CD90 and CD34. The numbers indicate the size (percentage) of the gated populations. CD81 expressions were shown in the right panel. All data are representative of at least three independent experiments.

To confirm the validity of our novel isolation method by using an alternative means of assessment, we next used FACS to confirm the immunological cellular landscape of the isolated layers (Fig. 2). Epithelial and hematopoietic cells were defined as EpCAM^+^ and CD45^+^ cells, respectively. FACS analysis revealed that EpCAM^+^ cells were rich in the epithelial layer that had been removed through de-epithelialization, and the proportion of epithelial cells gradually decreased in the histological preparations of the crypt and MM compartments (Fig. 2a). CD45^+^ cells were consistently present throughout the mucosa, but the submucosal microenvironment with the MM contained a relatively lower proportion of CD45^+^ cells (Fig. 2a). Even though the stratified isolation procedure extended the tissue processing time, cell viability in each layer was not critically decreased compared with that after traditional (unseparated whole-colon) cell-isolation methods (Supplemental Fig. 3a). Next, to clarify the methodological robustness, we assessed the cellular properties in independent experiments (Supplemental Fig. 3b). Importantly, the ratio of TCRγδ cells, recognized by their localization in the intraepithelial compartment, within CD45^+^ population was high in the epithelial layer, whereas CD4^+^ cell counts were higher in both the upper mucosa and cryptal layer^16^ (Supplemental Fig. 4). These results further proved the methodological validity of our stratified isolation strategies and indicate its versatility for further research.

Importantly, gp38^+^ cells, including most of the mesenchymal cells and lymphatic endothelial cells^17^, were preferentially distributed in the preparations from the upper mucosa compared with the MM with submucosal compartment (Fig. 2). Together, these results further indicate that this method achieved sufficient stratified isolation for spatial and phenotypic characterization of mesenchymal cells.

To precisely evaluate the colonic mesenchymal cellular populations by flow cytometry, we focused on gp38-expressing cellular populations. Specifically, we gated on gp38^+^ cells and then examined the expression of CD90 and CD34 (Fig. 2b). According to previous studies, CD90 and CD34 are expressed in the mesenchymal cells located in the crypt^8,18^; however, those markers were expressed in the mesenchymal cells in other microenvironments, including MM, which mostly consists of smooth muscle cells. Indeed, no previous study has examined the murine colon with exclusion of the submucosa, MM, and MEx; therefore, our results represent the first cellular profiling of the histologically different layers of the colonic mucosa.

Furthermore, CD34^–^ CD90^+^ mesenchymal cell populations, which contain myofibroblasts^8^, resided in the upper mucosa and MM but were less abundant in the crypt (Fig. 2b). The sub-epithelial compartment, crypt, and MM contained prominent CD34^+^ CD90^+^ and CD90^–^ mesenchymal cell populations but, comparably, those populations in the upper mucosal compartments were relatively small (Fig. 2c). In addition, recently CD81 expression in small intestinal cryptal mesenchymal cells has been reported^19^. Identically, our cryptal CD34^+^ cell highly expressed CD81 (Fig. 2c).

Our current evaluation uncovered enrichment of CD34^+^ mesenchymal cells in not only the crypt but also in the MM (Fig. 2c), indicating the need for cellular profiling of the cryptal region after the use of stratified isolation methods to separate the upper mucosa, crypt, and MM.

### Gene profiling of mesenchymal cells in the colonic crypt and luminal mucosa

To characterize crypt mesenchymal cells, we first examined the cell preparation obtained from mouse colon by use of the stratified isolation method. Because the upper mucosal compartment lacked a CD34^+^ population (Fig. 2c), to compare mesenchymal cells in the upper layers with those in the cryptal layers, we decided to further evaluate mesenchymal cells isolated by FACS sorting as CD45^−^ CD90^+^ gp38^+^^17^ and elucidated their gene expression profiles by using a gene microarray analysis. First, the two layers evaluated (cryptal layer and upper mucosal layer) showed identical expression of collagen genes (Col1a1 and Col1a2) (Supplemental Fig. 5a) and, identical to the FACS data, CD34 expression was dominant in the cryptal mesenchymal cells (Supplemental Fig. 5a). In addition, the genes related to cryptal niches—including Wnt5a, Wnt2b, Gli1, forkhead box l1 (Foxl1), and Sema3a^8,11,12,18,20^—were highly expressed in the cryptal mesenchymal cells (Supplemental Fig. 5a).

**Figure 3:**
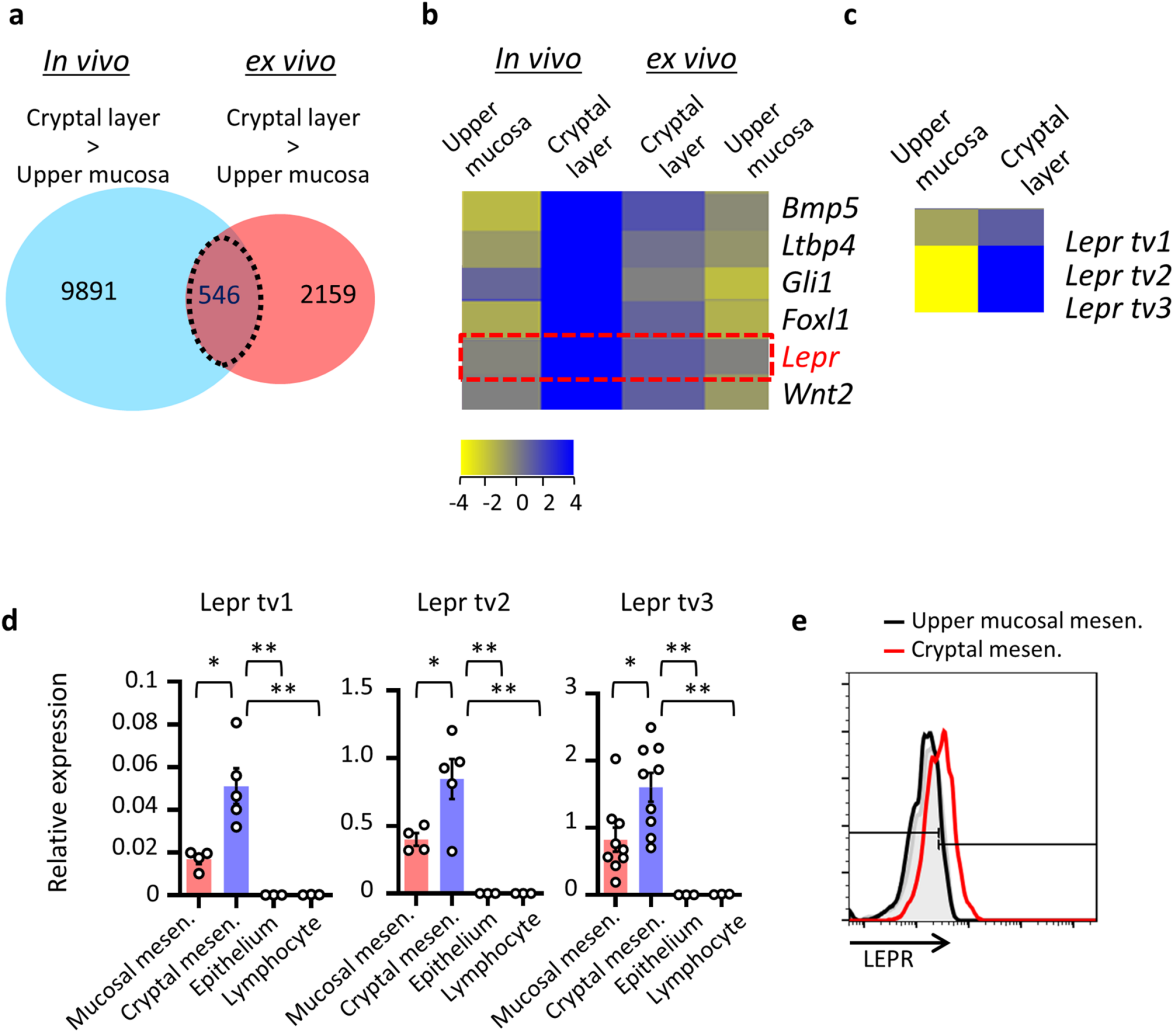
LepR expression in cryptal mesenchymal cells. (**a**–**c**) gp38^+^ mesenchymal cells isolated from the cryptal and upper mucosal layers underwent gene microarray analysis (n = 1). (**d**) Gene expression of leptin receptors in mesenchymal cells sorted from upper mucosa or crypt were examined with that of EpCAM^+^ epithelial cells and CD45^+^ lymphocytes by quantitative RT-PCR analysis. *P < 0.05. Each result was normalized against the expression of *Gapdh*. n.s. indicates not significant. (**e**) Leptin receptor expressions in upper mucosa or crypt were examined by flow cytometry. Gray histogram indicates isotype control. Data from a single representative experiment among three independent experiments are shown.

We then compared ex vivo-expanded mesenchymal cells, morphologically fibroblasts or myofibroblasts from the cryptal and luminal regions of the colonic mucosa (Supplemental Fig. 5b), with cells isolated in vivo by using the stratified isolation method and evaluated the genes expressed (Fig. 3a). Among those genes, 546 genes showed higher expression in the cryptal mesenchymal cells compared with the lumen regions of the colonic mucosa (Fig. 3a). The genes reported as highly expressed in cryptal mesenchymal cells, such as *Gli1*, *Foxl1*, *Bmp4*, and *Cd34*, are listed among the 546 genes (Fig. 3a,b) and top 30 genes in Table 1.

**Table 1:** Top 30 genes expressed in cryptal mesenchymal cells.

Rank	In vivo upper mucosa	In vivo cryptal layer	Ex vivo cryptal layer	Ex vivo upper mucosa	GeneSymbol	Description
1	− 0.1352253	10.349305	1.0953941	− 0.032011986	*Lepr*	Leptin receptor (Lepr), transcript variant 3, mRNA [NM_001122899]
2	− 1.7839279	9.163433	1.4320621	− 0.32384896	*Bmp5*	Bone morphogenetic protein 5 (Bmp5), mRNA [NM_007555]
	0	8.42976	0.75516224	− 1.6026034	*Bmp5*	Bone morphogenetic protein 5 (Bmp5), mRNA [NM_007555]
3	− 5.845847	8.349145	0	− 1.0225489	*Adamdec1*	ADAM-like, decysin 1 (Adamdec1), mRNA [NM_021475]
4	− 0.036146164	7.296133	1.1356153	− 0.10349226	*Pcdh12*	Protocadherin 12 (Pcdh12), mRNA [NM_017378]
5	0.8064318	7.193366	0	− 1.9055712	*Gli1*	GLI-Kruppel family member GLI1 (Gli1), mRNA [NM_010296]
6	− 0.31227016	7.19128	0	− 1.1451445	*Col15a1*	Collagen, type XV, alpha 1 (Col15a1), mRNA [NM_009928]
7	− 0.25886297	6.7288184	1.0249934	− 0.65438557	*Syne1*	Spectrin repeat containing, nuclear envelope 1 (Syne1), transcript variant 5, mRNA [NM_001347732]
8	0.40697193	6.3136005	0	− 1.2896981	*Lin7a*	Lin-7 homolog A (C. elegans) (Lin7a), transcript variant 1, mRNA [NM_001039354]
9	4.5943704	6.2623343	0	− 2.2177994	*Stxbp6*	Syntaxin binding protein 6 (amisyn) (Stxbp6), mRNA [NM_144552]
10	0	6.2549396	1.8562856	− 1.847434	*Grrp1*	Glycine/arginine rich protein 1 (Grrp1), mRNA [NM_001099296]
11	1.4523826	5.9267335	0	− 1.1692836	*Cadm3*	Cell adhesion molecule 3 (Cadm3), mRNA [NM_053199]
12	0.21515155	5.9007926	0	− 2.616846	–	UI-M-EX0-byj-o-02-0-UI.r1 NIH_BMAP_EX0 cDNA clone IMAGE: 5,719,129 5′, mRNA sequence [CB248850]
13	0.17255163	5.841345	0	− 1.2357674	*Agtr1b*	Angiotensin II receptor, type 1b (Agtr1b), mRNA [NM_175086]
14	2.6327944	5.812486	0	− 1.8589058	*Irf8*	Interferon regulatory factor 8 [Source:MGI Symbol;Acc:MGI:96395] [ENSMUST00000162775]
15	− 2.760572	5.737793	1.2296844	− 0.068935394	*Prdm6*	PR domain containing 6 (Prdm6), mRNA [NM_001033281]
16	− 1.346303	5.5345974	0.86455584	− 1.5482342	*Foxl1*	Forkhead box L1 (Foxl1), mRNA [NM_008024]
17	− 1.9157376	5.5081615	1.5659752	− 0.92323875	*Negr1*	Neuronal growth regulator 1 (Negr1), transcript variant 1, mRNA [NM_001039094]
18	− 2.9770136	5.4516544	1.3360395	− 0.24065113	*Bmp4*	Bone morphogenetic protein 4 (Bmp4), transcript variant 1, mRNA [NM_007554]
19	− 3.0122108	5.441319	0.7840228	− 0.26249528	*Bean1*	Brain expressed, associated with Nedd4, 1 (Bean1), transcript variant 1, mRNA [NM_001141922]
20	− 0.8304815	5.440546	0.45523453	− 0.61619663	*Ltbp4*	Latent transforming growth factor beta binding protein 4 (Ltbp4), transcript variant 1, mRNA [NM_175641]
21	− 4.281811	5.415837	0.8549876	− 0.26267695	*Tcf21*	Transcription factor 21 (Tcf21), mRNA [NM_011545]
22	0.30940437	5.3867254	0	− 1.0120187	*Cntn4*	Contactin 4 (Cntn4), transcript variant 1, mRNA [NM_001109749]
23	− 0.0352149	5.2685137	0.8375006	− 0.8934727	*Hcar1*	Hydrocarboxylic acid receptor 1 (Hcar1), mRNA [NM_175520]
24	− 1.1227269	5.265641	0.48159218	− 1.4391389	*Negr1*	Neuronal growth regulator 1 (Negr1), transcript variant 2, mRNA [NM_177274]
25	0.41755295	5.2641068	0	− 1.005084	*Tril*	TLR4 interactor with leucine-rich repeats (Tril), mRNA [NM_025817]
26	− 0.65405416	5.2553177	0.27284336	− 1.1687546	*Bend5*	BEN domain containing 5 (Bend5), transcript variant 1, mRNA [NM_026279]
27	0.6547599	5.2338214	0	− 1.1381359	*Ripply3*	Ripply transcriptional repressor 3 (Ripply3), mRNA [NM_133229]
28	0	5.189296	1.0285192	− 0.7719319	*Wnt2*	Wingless-type MMTV integration site family, member 2 (Wnt2), mRNA [NM_023653]
29	0	5.1865263	0.31024742	− 1.5084703	*Efcc1*	EF hand and coiled-coil domain containing 1 [Source:MGI Symbol;Acc:MGI:3611451] [ENSMUST00000160743]
30	1.6226387	5.186082	0	− 1.0252223	*Plac9a*	Placenta specific 9a (Plac9a), mRNA [NM_207229]

Results from in vivo- and ex vivo-expanded mesenchymal cells from the upper mucosa and crypt are shown. Top 30 genes of in vivo mesenchymal cells isolated from cryptal layers are shown.

We next focused on genes with “receptor/channel” function and speculated that these genes might be developmentally and functionally essential for cryptal mesenchymal cells^21^. Among the extracted genes, *Lepr*, *Hcar1*, *Vipr2*, *Agtra1b*, and *Adora1*, all showed higher expression in cryptal mesenchymal cells based on the gene array (Fig. 3b and data not shown). The ligands of these receptors are leptin, lactate, vasoactive intestinal peptide, angiotensin II, and adenosine, respectively^22–25^, and some of the receptors (e.g., *Lepr*, *Hcar1*, and *Adora1*) are involved in mucosal homeostasis^22,26,27^. Among those receptors, leptin is an important adipokine, derived from adipose tissues, and is involved in obesity, immune responses, and inflammation^28,29^. Several receptor isoforms exist^23,30^, and indeed trans-variants 1 through 3 were highly expressed in cryptal mesenchymal cells (Fig. 3c). Collectively, gene profiling of the mesenchymal cells in the colonic crypt and luminal mucosa yielded previously unknown cryptal mesenchymal cell-receptors, which—especially in the case of receptors for leptin—might be important for mesenchymal cell function in crypts. To confirm this issue, we used qRT-PCR to further examine the expressions of the receptors (Fig. 3d). We used our stratified cell isolation method to obtain isolated cells that were CD45^−^ EpCAM^−^ and positive for the general mesenchymal cell markers, CD90 and gp38^17^, from the luminal mucosa and crypts. The expression levels of leptin receptors (LepRs) 1 through 3 were significantly higher in the cryptal CD90^+^ gp38^+^ mesenchymal cells than the luminal mesenchymal cells compared with other cellular components (Fig. 3d). In addition, surface leptin receptor expressions were higher in crypt mesenchymal cells (Fig. 3e).

These evidences possibly indicate the critical role of intrinsic leptin for functional regulation of mesenchymal cells, especially in crypts. Intriguingly, when cryptal mesenchymal cells were isolated from germ-free mice and examined for the expression of LepR, the degree of the receptor expression was unaltered regardless of the presence or absence of commensal microbiota (Supplemental Fig. 6). This result indicated the de novo programmed expression of the LepR in mesenchymal cells in the crypt and the expressions were dispensable of signaling from commensal bacteria.

### Wnt2b expression is retained in cryptal mesenchymal cells through leptin and LepR

Because the receptors for leptin were dominantly expressed in the colonic cryptal mesenchymal cells (Fig. 3), we further evaluated the biological role of leptin and its receptors in terms of the function of cryptal mesenchymal cells. Cryptal mesenchymal cells are considered to be important in mucosal regeneration and homeostasis^18^; therefore, we examined whether the leptin system is involved in the production of cryptal niche factors.

**Figure 4:**
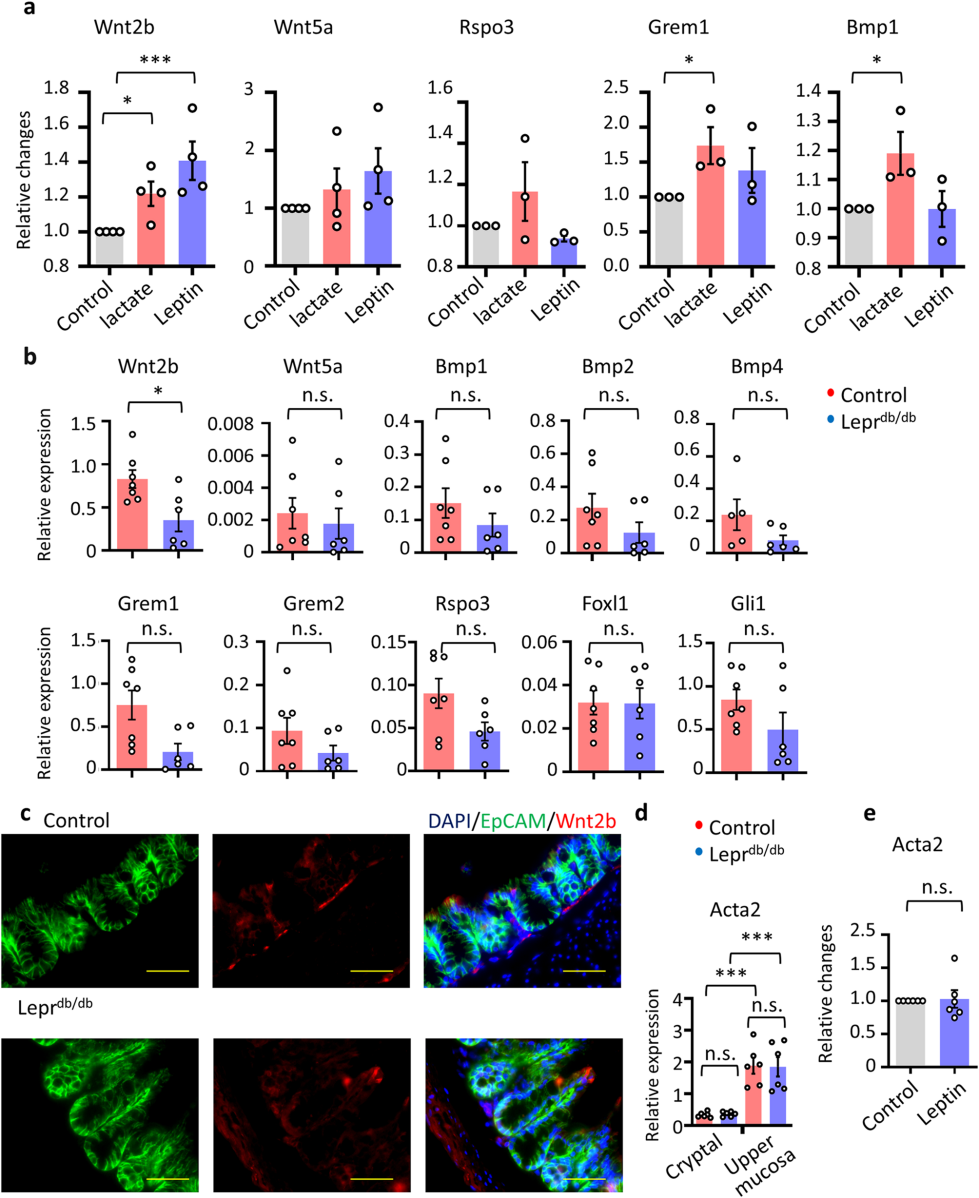
Wnt2b expression is retained in cryptal mesenchymal cells through the LepR. (**a**) ex vivo cryptal mesenchymal cells were stimulated by leptin or lactate and the expression of cryptal stem niches genes was examined. Relative changes normalized by normalized against the expression of *Gapdh* were shown. Each dot represents an individual subject (n = 3 or 4) (**b**,**c**) Cryptal mesenchymal cells were sorted from control and *Lepr*^*db/db*^ mice. (**b**) The expression of cryptal stem niches genes was examined by qRT-PCR analysis. Each result was normalized against the expression of *Gapdh.* Each dot represents an individual subject (n = 6 or 7). (**c**) Colon tissues of control and *Lepr*^*db/db*^ mice were stained by Wnt2b and EpCAM. Representative data from 3 mice are shown. Scale bar, 50 μm. (**d**) The expression of *Acta2a* was examined by qRT-PCR analysis. Each result was normalized against the expression of *Gapdh.* Each dot represents an individual subject (n = 6). (**e**) ex vivo cryptal mesenchymal cells were stimulated by leptin and the expression of *Acta2* was examined (n = 6). Data are shown as means ± SEM; *P < 0.05, ***P < 0.01. n.s. indicates not significant.

Lactate has been reported to have a regulatory function in mucosal regeneration and mobility^24,27^. After intestinal injury due to either radiation or chemotherapy, lactate stimulation of receptors promotes production of niche factors, including WNTs, and stimulates stem cell proliferation^27^. We thus first evaluated whether leptin stimulation resulted in the production of crypt niche-related factors, such as *Wnt2b*, *Wnt5a*, *Rspo3*, *Grem1*, and *Bmp1*, by comparing the results before and after leptin stimulation. We found that Wnt2b was indeed induced after leptin stimulation, thus indicating the potential involvement of leptin in mucosal protection and repair from injury (Fig. 4a).

We next evaluated LepR-deficient mice (*Lepr*^*db/db*^) regarding their gene expression levels of representative intestinal stem cell niche factors derived from cryptal CD90^+^ gp38^+^ mesenchymal cells, including WNTs, BMPs, GREMs, or transcription factors (e.g., *Gli1* and *Foxl1*) (Fig. 4b)^23^. The expression of BMPs, Grem1, Grem2, Rspo3, transcription factors (*Gli1* and *Foxl1*), and *Wnt5a* in cryptal mesenchymal cells was not significantly different between *Lepr*^*db/db*^ and control strains (Fig. 4b)^11,20^. In agreement with the stimulation analysis (Fig. 4a), the expression of *Wnt2b* was significantly reduced in the cryptal mesenchymal cells from LepR-deficient *Lepr*^*db/db*^ mice compared with controls, indicating the importance of LepR signaling for Wnt2b expression (Fig. 4b).

Importantly, histological analysis revealed that a strong Wnt2b signal was present in the cryptal region of control mice (Fig. 4c), whereas this signal was much weaker in the *Lepr*^*db/db*^ mice. These findings are consistent with the gene expression data in Fig. 4b.

Recently it has been indicated that LepR^+^ mesenchymal cells found in the bone marrow are α-smooth muscle actin (Acta2)^+^ myofibroblasts and are considered as a source of osteoblasts and adipocytes^31,32^. We examined the expression of Acta2 in the cryptal and upper mucosal CD90^+^ gp38^+^ mesenchymal cells isolated by our stratified cell isolation methods. Acta2 expression was low in the cryptal mesenchymal cells, indicating these cells are not myofibroblasts but fibroblasts (Fig. 4d). In addition, Acta2 expression levels in WT mice were comparable to those in the cells from LepR-deficient mice (Fig. 4d). In addition, in vitro stimulation with leptin of CD90^+^ gp38^+^ mesenchymal cells isolated from LepR-deficient mice caused no change in Acta2 expression (Fig. 4e), indicating the indispensable role of leptin signals in Acta2 expression and myofibroblast development in the steady state (Fig. 4d).

These results collectively indicate the involvement of leptin in the production of Wnt2b, which is essential for the survival and proliferation of intestinal epithelial cells, for gut homeostasis after mucosal inflammation, indicating that leptin signals through the colonic cryptal mesenchymal cells are possibly involved in mucosal protection.

### Leptin suppresses intestinal inflammation

**Figure 5:**
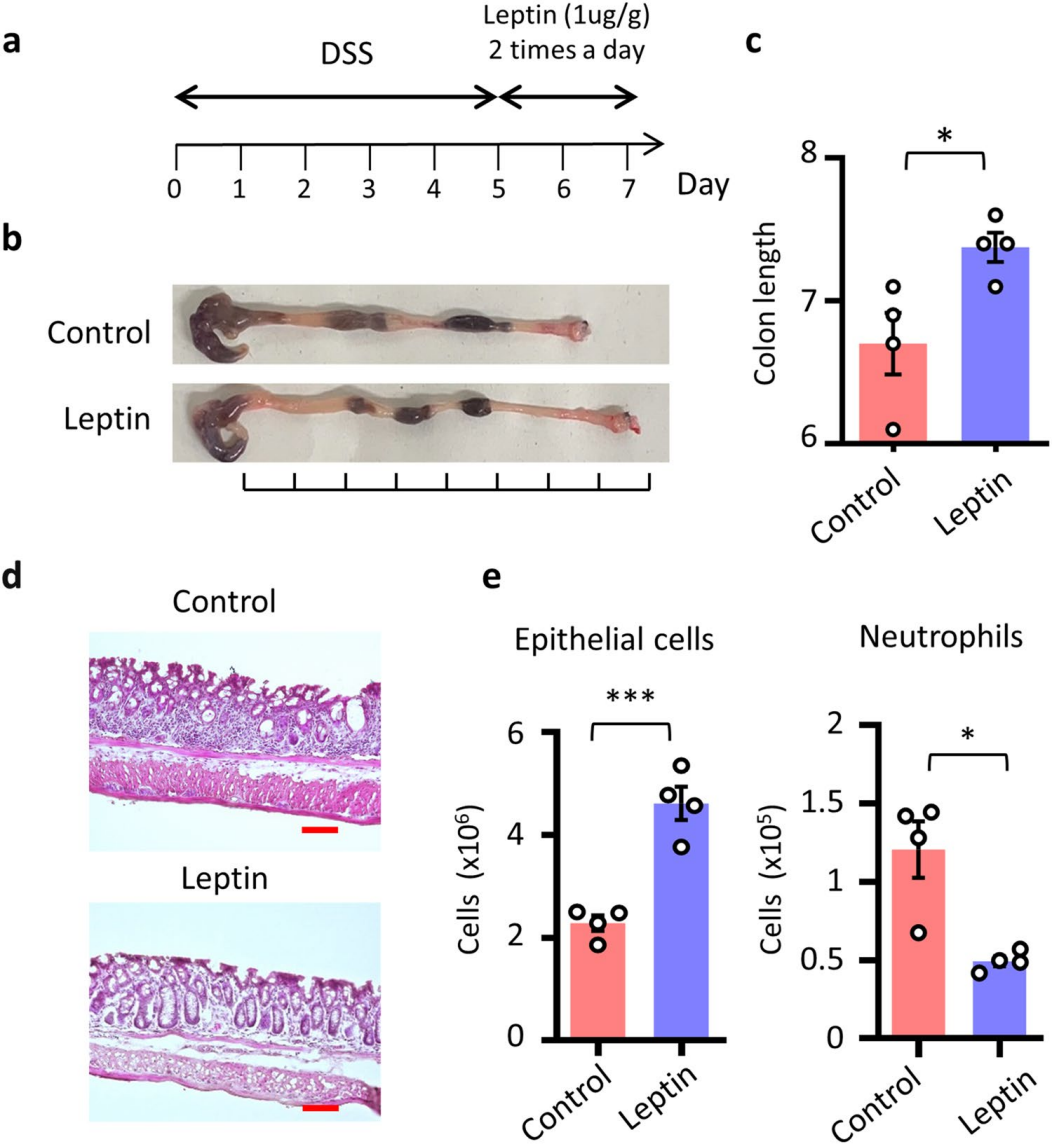
Leptin administration protects intestinal inflammation. (**a**) Time course of this experiment. Mice underwent DSS treatment, and leptin (1 μg/g) was administered twice daily under fasting conditions to limit intrinsic leptin. (**b**–**d**) Representative images and colon length are shown (n = 4 per group). Scale bar, 100 μm. (**e**) Whole colon cells are isolated and the cell number of EPCAM^+^ epithelial cells and infiltrated CD11b^+^ Gr-1^+^ neutrophils in the colon were shown. *P < 0.05, ***P < 0.01. All data are representative of two independent experiments.

Our present study demonstrated that intrinsic leptin signals in cryptal mesenchymal cells are involved in the expression of *Wnt2b*, which is considered to be important for mucosal regeneration^12^. To begin with, we evaluated the protective effect of leptin in mucosal damage. We administered leptin to mice with dextran sodium sulfate (DSS)-induced colitis mice under conditions of reduced endogenous leptin signaling due to food deprivation after DSS administration^33^ (Fig. 5a): 1 μg/g (body weight) of leptin was administered intraperitoneally during the fasting period^28^ (Fig. 5a). Mice that received leptin showed reduction of colon shortening, which is one of the markers in the assessment of colonic inflammation (Fig. 5b,c). In addition, crypt morphology was revealed through hematoxylin-and-eosin staining of colonic sections, and FACS analysis was performed to evaluated epithelial cell number and neutrophil accumulation throughout the colon (Fig. 5d,e). These analyses revealed that the epithelial layer and crypt structure were similar between the leptin-treated and control groups but that the accumulation of neutrophils was decreased after leptin administration (Fig. 5d,e). These results suggested the important roles of leptin in the mucosal protection.

### Intrinsic leptin controls mucosal homeostasis

**Figure 6:**
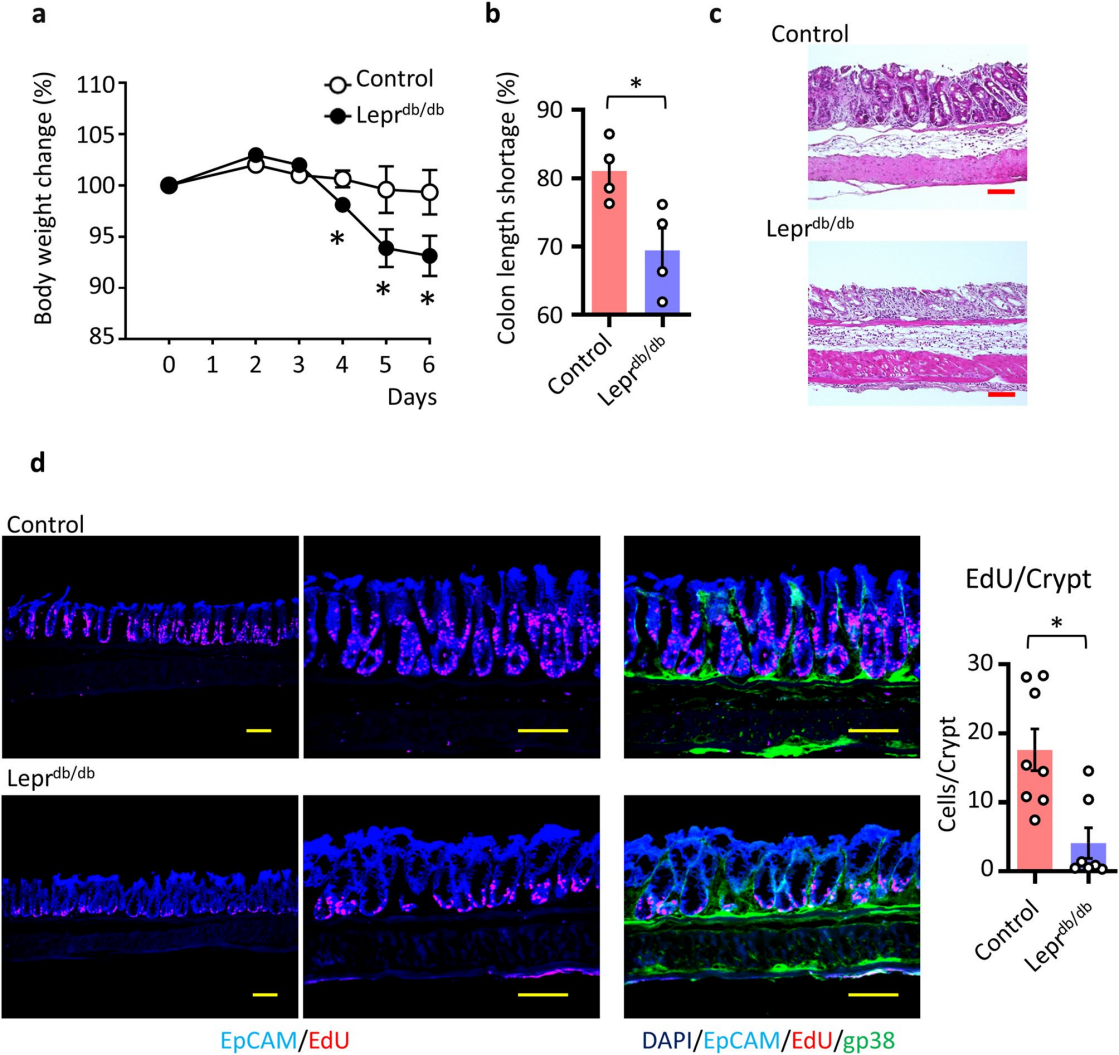
Intrinsic leptin controls mucosal healing through LepR^+^ cryptal mesenchymal cells. (**a**) Body weight change (n = 4 per group). *P < 0.05. (**b**) Ratio of control and DSS treated mice colon length are shown. Data are shown as means ± SEM; *P < 0.05. (**c**) H and E staining of colon on day 6 of DSS treatment is shown. Scale bar, 100 μm. (**d**) Immunohistochemical analysis of DSS-treated mice on day 6. EdU was administered intraperitoneally 3 h before euthanasia. Tissues were stained with DAPI, EpCAM, and gp38 with detection of proliferated EdU^+^ cells. Representative data from 4 mice are shown. Scale bar, 100 μm. EdU^+^ cells counted in per crypt were shown. All data are representative of two independent experiments.

Wnt2b is involved in mucosal epithelial and cryptal regeneration during colitis^8^. Therefore, we sought to elucidate whether the leptin–cryptal mesenchymal pathway plays a critical role at the colonic mucosal epithelial barrier. To elucidate intrinsic leptin and LepR signaling in mucosal homeostasis, we exposed *Lepr*^*db/db*^ and littermate control mice to DSS-induced colonic inflammation and examined them for sensitivity to colitis development (Fig. 6a,b). DSS-treated *Lepr*^*db/db*^ mice showed more severe body weight loss and shortened colon length, representing well-known disease symptoms^34^ (Fig. 6a,b). In addition, histological assessment revealed that the mucosa of DSS-treated *Lepr*^*db/db*^ mice showed necrosis and epithelial damage with erosion, loss of epithelial cells, and crypt ectasias (Fig. 6c). Epithelial damage involved deeper colonic crypts in *Lepr*^*db/db*^ mice compared with control mice (Fig. 6c).

Epithelial morphology and the proportion of Ki67^+^ proliferated cells (indicative of epithelial turnover) in the steady state did not differ between *Lepr*^*db/db*^ and control mice (Supplemental Fig. 7). However, during colitis, the proliferative zone of 5-ethynyl-2′-deoxyuridine (EdU)-stained (red cells) in the crypt was significantly reduced compared with that of control mice (Fig. 6d). These results collectively indicated the importance of Wnt2b from cryptal mesenchymal cell populations through intrinsic hormones, especially leptin, for the maintenance of mucosal homeostasis. Thus, our novel stratified layer isolation strategies uncovered novel and unique characteristics of cryptal mesenchymal cells, sensing intrinsic leptin for controlling mucosal homeostasis.

## Discussion

Mesenchymal cells in gut play important roles in the orchestration of immune cells for lymphoid organogenesis, regulation of immune responses, and regeneration of the epithelial barrier^5^. Epithelial regeneration is an important process for mucosal homeostasis and is consistent with consequent processes such as coverage of denuded compartments, and stem cell proliferation and development of a functional epithelial layer, including columnar epithelial cells, secretory type cells (e,g., goblet cells and Paneth cells), and enteroendocrine cells^17^. Cytokines involved in epithelial development, such as EGF and WNTs, are produced from Paneth cells and mesenchymal cells, located in the crypt of the small or large intestines^5,9^. Especially in the colon, which lacks Paneth cells, mesenchymal cells have major roles in crypt and epithelial development and homeostasis^11^.

Accumulated evidence has revealed the involvement of mesenchymal cells, such as fibroblasts and myofibroblasts, which are located in the cryptal or pericryptal regions, in the regulation of epithelial–immunological homeostasis^17^. LepR^+^ mesenchymal cells have been shown to possess specific functions in other organs, such as bone marrow^31,32^; however, LepR signaling is not involved in the certain function of this specific cell populations^31^. In colon, recent single-cell RNA-Seq analysis revealed that at least four subsets of fibroblasts are present in the mice colon^4,35^. We have not evaluated whether LepR^+^ pericryptal mesenchymal cells are a specific sub-population in the CD34^+^ population. Future studies will have to define whether LepR^+^ cells are a specific sub-population of fibroblasts.

In this context, a unique type of interstitial cell, with long projections, or telopodes, has recently been found; these cells are called telocytes and are located surrounding crypts^36^. Telocytes express CD34 but not c-kit, unlike another type of intestinal crypt cell, Cajal cells, which are the pacemakers of peristaltic movement^5^. Cryptal mesenchymal cells obtained by the stratified isolation protocol contained crypt cells that expressed CD34 and Foxl1, hallmarks of fibroblasts and telocytes, thus supporting the methodological validity and reliability of the present study (Fig. 3). Conditional deletion of Wnt production from Foxl1^+^ cells led to the abolishment of stem and transit-amplifying cell proliferation^20^. However, our finding revealed that other CD34^+^ cell populations are also located in the other layers, such as the epithelial layers and MM, in the colon (Fig. 2). The past studies seemed to not consider the potential for the contamination of smooth-muscle cells and mesenchymal cells located in MM, submucosa, and MEx, because whole-cell isolation methods with digestive enzymes generally were used^8,18^. In fact, CD34^+^ CD90^+^ mesenchymal cells exist in the MM fraction as well (Fig. 2)^8,18^. Therefore, here we have shown the methodological significance and compelling utility of our stratified isolation method in designing experiments that analyze various histologically distinct parts of the colonic mucosa (e.g., lumen to crypt).

From our analysis of cryptal mesenchymal cells, we learned the beneficial feature of leptin in promoting epithelial turnover in the inflammatory state. Even though the current histological analysis of the intestine showed no modification in the architecture of the epithelium in *Lepr*^*db/db*^ mice (Supplemental Fig. 7)^37^, a recent study has indicated that physiological intestinal barrier functions are weakened during decreased leptin signaling^38^. In particular, hyperglycemia in *Lepr*^*db/db*^ mice caused disruption of tight junction integrity, leading to enhanced influx of luminal molecules, increased electrical current across the epithelial layer, and heightened susceptibility to enteric infection^38^. In addition, the induction of hyperleptinemia through subcutaneous administration of exogeneous leptin increased the mucosal barrier and lessened damage severity^39^, indicating the possible involvement of leptin–mesenchymal pathways in novel regulatory pathways of mucosal protection. However, the regulation of epithelial regeneration via indirect pathways of cryptal mesenchymal cells with alternative leptin pathways might be involved in the phenotype of *Lepr*^*db/db*^ mice. For example, leptin is involved in gastric motility as well as in the migration of CCR7^+^ dendritic cells and differentiation of T cells (e.g., Th1/Th2 cytokine balance and Th17 differentiation)^26,40–42^. We found that the expression level of LepRs was higher in colonic cryptal mesenchymal cells compared with epithelial cells and CD45^+^ hematopoietic cells (Fig. 3d). Therefore, we emphasize a critical role of leptin and its receptor interaction on colonic cryptal mesenchymal cells; however, we cannot ignore the possible involvement or crosstalk of leptin to or with other cellular populations.

Leptin has also been shown to be associated with obesity, a form of chronic inflammation, considered in the etiopathogenesis of intestinal inflammation^43^. Obesity is the hallmark phenotype of *Lepr*^*db/db*^ mice, and it may be that obesity causes delayed mucosal regeneration. However, obesity is a proinflammatory state in mice and humans, and adipose tissue produces inflammatory cytokines; therefore, these cytokines may contribute to delayed epithelial growth via another mechanism^44,45^.

For example, various adipocytokines (e.g., adiponectin, apelin, chemerin, leptin) are secreted from adipocytes, but in obesity, the regulation of the production of these adipocytokines is aberrant due to enlarged adipocytes^46,47^. Furthermore, inflammation-associated pathological alterations of adipocytes in mesenteric fat have previously been reported^46^, as have reduced expression levels of adiponectin in colitis mice^44,45^. Our group and others have reported on the functional transition of mesenchymal fat (e.g., increase of TNF-α and reduction of adipokines) and its effects on immune and epithelial integrity in colitis^45,48^. Adiponectin has an anti-inflammatory reaction against TNF-α pathways, such as NF-κB activation^49^. Indeed, we found a reduction of leptin expression in the adipose tissue of colitis (Supplemental Fig. 8). The use of the stratified isolation method opens new areas of investigation into the molecular and cellular interactions between anatomically and histologically different colonic crypt cells and adipose tissues.

Our findings from gene profiling revealed that several receptors in addition to LepR might contribute to the functioning of crypt mesenchymal cells (Fig. 3, Table 1). In particular, receptors for lactate and adenosine have been reported in regard to their regulatory function in mucosal regeneration and mobility^22,24,27^. In addition, lactate produced from *Bifidobacterium* and *Lactobacillus spp*. stimulates the lactate receptor expressed in the Paneth cells and fibroblasts of the small intestine^27^. After intestinal injury due to radiation and chemotherapy drugs, lactate stimulation of receptors promotes Wnt3 production and stimulates stem cell proliferation^27^. Further investigation to examine and elucidate the corporative regulatory systems through the multiple receptors that exist in the crypt region is warranted.

Taking together all of these previous and current findings, our new and reliable stratified isolation method revealed the unique characteristics of various types of mesenchymal cells isolated from different histological locations in the colonic mucosa and disclosed a novel pathway for intrinsic control of leptin-mediated mucosal protection (Supplemental Fig. 9). Our initial findings from using this stratified method for isolating mesenchymal cells yield insight into mechanisms of epithelial regeneration and treatment strategies for intestinal inflammation.

## Materials and methods

### Mice

Male C57Bl/6 mice, BKS.Cg- + *Lepr*^*db*^*/* + *Lepr*^*db*^*/Jcl* (*Lepr*^*db/db*^ mice), and BKS.Cg-^*m*+*/m*+^  + *Jcl* (control litter) mice (age, 7 to 10 weeks) purchased from CLEA (Tokyo, Japan) were used throughout the study. Twist2-Cre and Rosa26-tdTomato mice were purchased from Jackson Laboratory (Bar Harbor, ME, USA), Col1a2-GFP mice were a gift, as previously described^50^. Colitis was induced by using 2.25% DSS, and body weight was measured as described previously^51^. For the leptin administration, mice 1ug/g body weight of leptin or PBS as control were administered intraperitoneally during fasting period for two days according to the previous publication^33^.

All mice were maintained under specific-pathogen-free (SPF) conditions at the experimental animal facility of the Institute of Medical Science, The University of Tokyo and Chiba University. All experiments were approved by the Animal Care and Use Committee of the University of Tokyo (PA17-89 and PA17-96) and Chiba University (A30-112)**.** All procedures were performed in accordance with relevant guidelines and regulations.

### Stratified isolation of mouse colon tissues

Colons were collected from mice, washed with PBS, opened vertically, and shaken 40 times with 25 ml of PBS to remove fecal contents and mucus. These processes were repeated twice. Harvested colon tissues were incubated in cell recovery solution (Corning, High Wycomb, UK)^45,52^ for 1 h on ice (Supplemental Fig. 1). Then, the epithelial and sub-epithelial cells were scraped off gently by using the round part of a J-shaped microtweezer until the honeycomb structures of the mucosal lamina propria were seen under microscopy (model S9D, Leica, Wetzlar, Germany); simultaneously MEx was peeled off (Supplementary Video). After the MEx was peeled from the colon tissue, each colonic layer was isolated as follows. To isolate the upper mucosal cells, upper mucosa was scraped gently away by using the round part of J-shaped microtweezers and scraper (Supplemental Fig. 1 and Supplementary Video). Then, the colonic tissues were turned over, and the MM and submucosal compartments were peeled off. The layer remaining is consistent with the cryptal layer (Supplemental Fig. 1 and Supplementary Video). All procedures are available in the technical video.

### Cell collection and FACS analysis

Mononuclear cells were isolated from each colonic layer, according to slight modifications from previously described protocols^51^. Briefly, epithelium was dissociated by using a cell recovery solution (Becton Dickinson, San Jose, CA, USA) as described previously^52^, and the isolated de-epithelialized layers of whole colon were digested in 1.25 mg/ml collagenase at 37 °C (WAKO, Takasaki, Gunma, Japan) for 80 min^51^. The collected mononuclear cells were subsequently filtered through a 100-μm cell strainer (BD Biosciences, San Jose, CA, USA) and examined further.

For flow cytometric analysis, cells were incubated for 5 min with anti-CD16/32 antibody (5 μg/ml; Fc Block, BD Pharmingen, Franklin Lakes, NJ) and stained for 30 min at 4 °C with 7-AAD and fluorescent-labeled antibodies specific for CD11b, CD34, CD45, CD81, CD90.2, EpCAM, gp38, Gr-1, and TER119 (BioLegend, San Diego, CA, USA) and leptin receptor (Abcam, Cambridge, MA, USA). Cells were analyzed by flow cytometry (FACS Canto II, Becton Dickinson) or Attune NxT Acoustic Focusing Flow Cytometer (Thermo Fisher Scientific, MA, USA).

To expand mesenchymal cells ex vivo, CD90^+^ gp38^+^ CD45^−^ EpCAM^−^ cells were isolated by using a FACS Aria III (Becton Dickinson) and cultured with 10 ng/ml of EGF (PeproTech, Rocky Hill, NJ, USA). Cells were stimulated by adding 200 ng/ml leptin (Sigma, St. Louis, MO, USA) and 5 mM lactate (Sigma) to the media and incubating overnight; stimulated cells were collected for further analysis^27,53^.

### Quantitative RT-PCR and gene microarray analyses

Total RNA was prepared by using TRIZOL (Thermo Fisher Scientific, Waltham, MA, USA) and reverse-transcribed by using Superscript IV VILO (Thermo Fisher Scientific), as described previously^54^. Quantitative RT-PCR analysis used the LightCycler 96 (Thermo Fisher Scientific) and the Universal Probe Library (Roche, Basel, Switzerland). The primer set are listed in Table 2.

**Table 2:** Primer lists for the experiments.

	Forward 5′–3′	Reverse 5′–3′
Gapdh	TGTCCGTCGTGGATCTGAC	CCTGCTTCACCACCTTCTTG
Wnt2b	CCGGGACCACACTGTCTTT	GCTGACGAGATAGCATAGACGA
Wnt5a	ACGCTTCGCTTGAATTCCT	CCCGGGCTTAATATTCCAA
Rspo3	TCAAAGGGAGAGCGAGGA	CAGAGGAGGAGCTTGTTTCC
Grem1	GACCCACGGAAGTGACAGA	CCCTCAGCTGTTGGCAGTAG
Grem2	GAGGAGAGGGACAGGGAGAC	AGCGAGAGCTTCCAGAACAT
Bmp1	GAAGGCCATCCATCAAAGC	CCACTAGTGCCCTGACCAC
Bmp2	AGATCTGTACCGCAGGCACT	GTTCCTCCACGGCTTCTTC
Bmp4	GAGGAGTTTCCATCACGAAGA	GCTCTGCCGAGGAGATCA
Gli1	TGGAGGTCTGCGTGGTAGA	TTGAACATGGCGTCTCAGG
Foxl1	CCATGAAGAAGGGACAAAGC	CCACCGGGGAGTCCTAAG
Lepr1	GTTCCAAACCCCAAGAATTG	TGATTCTGCATGCTTGGTAAA
Lepr2	CATTTCCGCTTCAATATCAGG	CCAGCAGAGATGTAGCTGAGAC
Lepr3	GTTCCAAACCCCAAGAATTG	GACTTCAAAGAGTGTCCGTTCTC
Leptin	CAGGATCAATGACATTTCACACA	GCTGGTGAGGACCTGTTGAT
Hcar1	GGCTGAGAAAAGCGGTATGA	TCGTTAACTCTCTCCGAGCTAGA
Vipr2	GGCCTGGTGGTAGCAGTTC	TCGCCATCTTCTTTTCAGTTC
Adora1	GTCAAGATCCCTCTCCGGTA	CAAGGGAGAGAATCCAGCAG
Agtr1b	CGCCAGCAGCACTGTAGA	GGGGGTGAATTCAAAATGC

Sequence of primers for the experiments are listed.

Microarray analysis was performed as in our previous report^54^. Briefly, mononuclear cells were sorted, and total RNA was extracted from them by using TRIZOL (Thermo Fisher Scientific). cRNA was hybridized with DNA probes on a GeneChip Mouse Genome array (Agilent, Santa Clara, CA, USA). Data were analyzed by using GeneSpring software (Silicon Genetics, Redwood City, CA, USA).

### Histological analysis

Hematoxylin and eosin staining (Muto Pure Chemicals, Tokyo, Japan) and immunohistochemical analysis were performed as previously described^51^. For immunohistochemistry, samples of large intestine were fixed in 4% paraformaldehyde (Wako) and incubated in 20% sucrose. The tissues were embedded in OCT compound (Sakura Fine Technical Company, Tokyo, Japan). Tissue sections were stained with anti-mouse EpCAM (Biolegend, San Diego, CA, USA), anti-mouse gp38 (Biolegend), Ki67 (Biolegend), and Wnt2b (Abcam). Anti-rabbit IgG conjugated with Alexa 488 (Thermo Fisher Scientific) served as a secondary antibody.

### EdU analysis

At 3 h before euthanasia, mice were intraperitoneally injected with 100 µg/200 µl of EdU (Thermo Fisher Scientific). EdU staining was performed in accordance with the manufacturer’s instructions.

### Statistical analysis

Statistical analysis was performed by using unpaired, two-tailed Student’s *t*-tests (Prism 8, GraphPad Software, San Diego, CA, USA). A *P* value < 0.05 was considered to be statistically significant. Data are reported as mean ± SEM (error bars in figures).

## Supplementary information

Supplementary Video 1.

Supplementary Figures.
