# Supplementary material for: Stratified layer analysis reveals intrinsic leptin stimulates cryptal mesenchymal cells for controlling mucosal inflammation
Source: Sci Rep. 2020 Oct 27;10:18351. doi: 10.1038/s41598-020-75186-3 (PMC7591933; doi:10.1038/s41598-020-75186-3)
Supplement: Supplementary file 2 — Supplementary Figures. [file 41598_2020_75186_MOESM2_ESM.pdf]

Stratified Layer Analysis Reveals Intrinsic Leptin Stimulates Cryptal Mesenchymal Cells for Controlling Mucosal Inflammation

Seiichi Matsumura[1–3#], Yosuke Kurashima[1,2,4,5,7#*], Sayuri Murasaki[2], Masako Morimoto[1], Fujimi Arai[2], Yukari Saito[1], Nana Katayama[2], Dayoung Kim[2], Yutaka Inagaki[6], Takahiro Kudo[3], Peter Ernst[5,7,8], Toshiaki Shimizu[3], Hiroshi Kiyono[2,4,5]

1. Department of Innovative Medicine, Graduate School of Medicine, Chiba University, Chiba, 260-8670, Japan
2. Department of Mucosal Immunology, The University of Tokyo Distinguished Professor Unit, The Institute of Medical Science, The University of Tokyo, Tokyo, 108-8639, Japan
3. Department of Pediatrics, Juntendo University Faculty of Medicine 2-1-1 Hongo, Bunkyo-ku Tokyo, 113-8421, Japan
4. International Research and Development Center for Mucosal Vaccines, The Institute of Medical Science, The University of Tokyo, Tokyo 108–8639, Japan
5. Division of Gastroenterology, Department of Medicine, CU-UCSD Center for Mucosal Immunology, Allergy and Vaccines (CU-UCSD cMAV), University of California, San Diego, CA 92093-0956, USA
6. Center for Matrix Biology and Medicine, Graduate School of Medicine, Tokai University, Kanagawa, Japan Department of Regenerative Medicine, Tokai University School of Medicine, Isehara, Japan; Center for Matrix Biology and Medicine, Tokai University Graduate School of Medicine, Isehara, Japan
7. Division of Comparative Pathology and Medicine, Department of Pathology, University of California San Diego, San Diego, CA 92093-0956, USA
8. Center for Veterinary Sciences and Comparative Medicine, University of California, San Diego, CA 92093-0956, USA

#Authors contributed equally
*Corresponding author

*Correspondence should be addressed to

Yosuke Kurashima, PhD
Department of Innovative Medicine,
Graduate School of Medicine, Chiba University, Chiba, Japan
1-8-1 Inohana, Chuo-ku, Chiba-shi, Chiba, 260-8670, Japan
Tel: +81-43-226-2848, Fax: +81-43-226-2183
E-mail: yosukek@chiba-u.jp/yosukek@ims.u-tokyo.ac.jp

Supplemental Figure 1

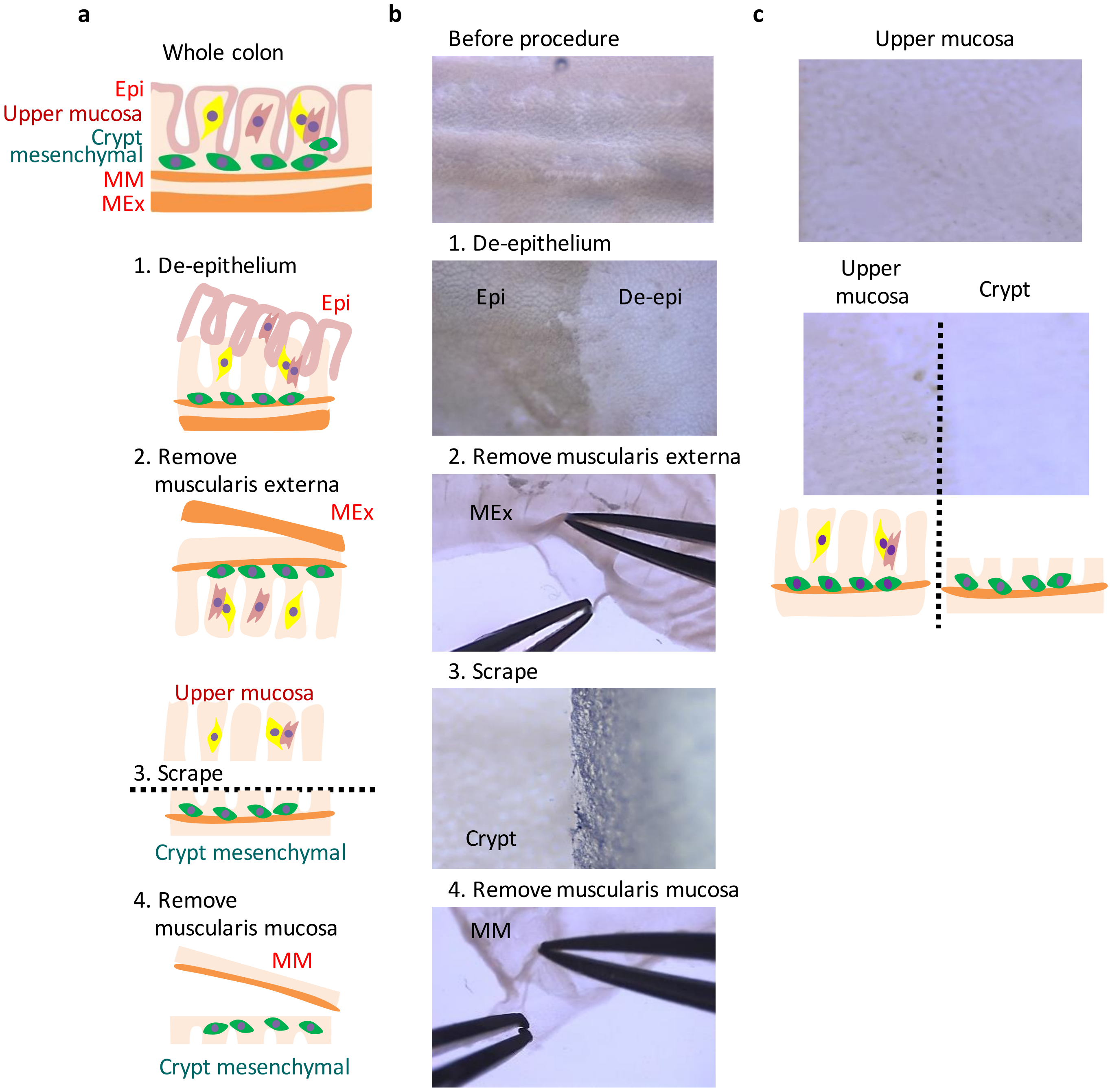

Supplemental Figure 1. Isolation scheme for collecting cryptal basal mesenchymal cells.

(a) The scheme for stratified isolation is shown. (b) Images of colon tissues after each procedure of the stratified isolations are shown. (c) Representative pictures of before and after isolation of the upper mucosa are shown.

Supplemental Figure 2

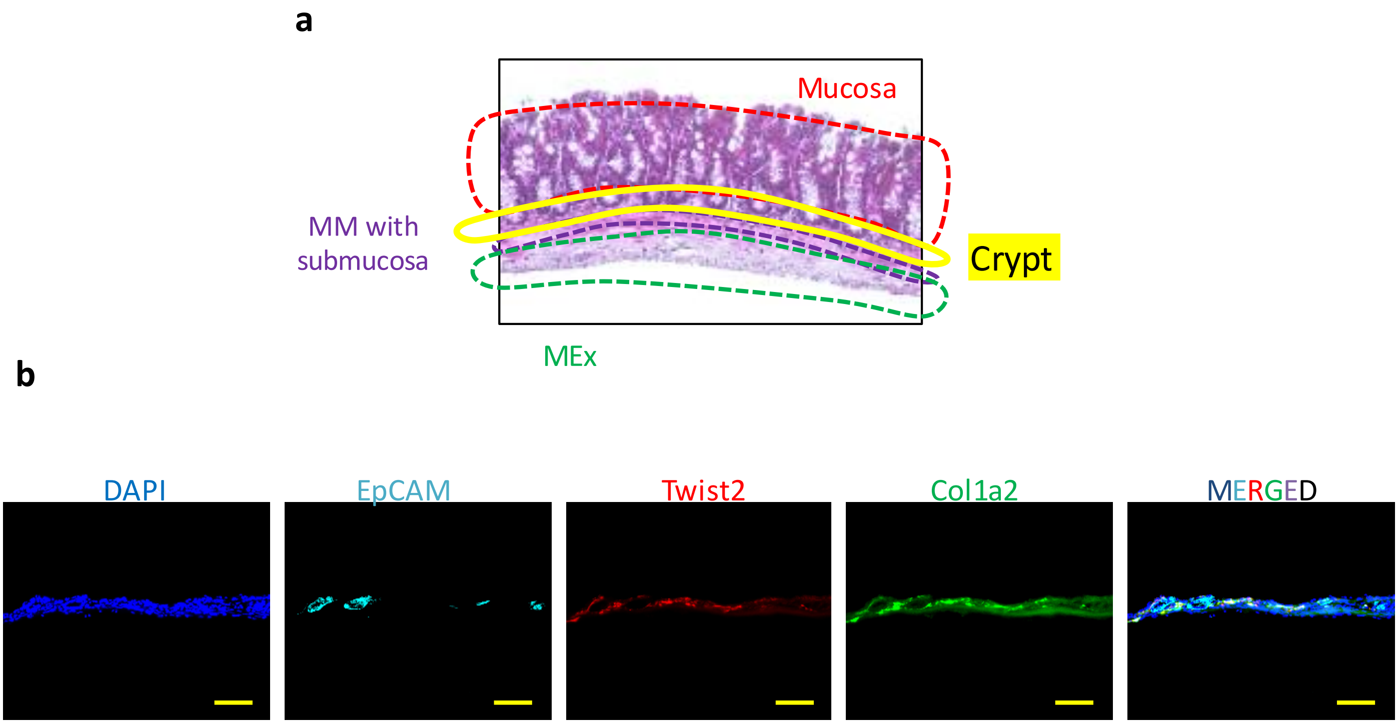

Supplemental Figure 2. Additional independent experimental picture for cryptal basal tissues.

(a) The scheme for stratified isolation is shown. (b) Crypt region of Twist2-Cre tdTomato x Col1a2 GFP mice is shown from independent experiment. Bar, 100 µm.

Supplemental Figure 3

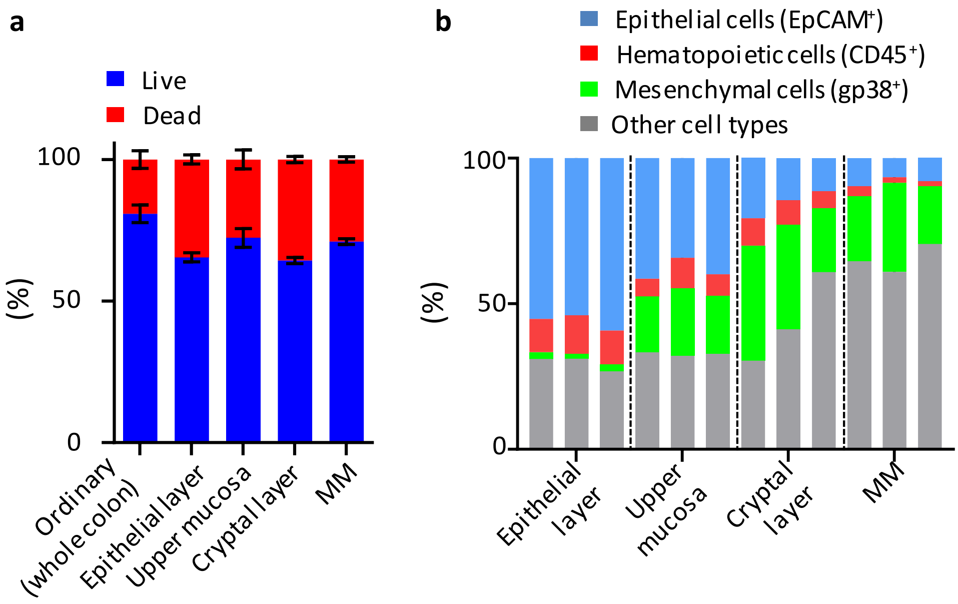

Supplemental Figure 3. Validation of stratified cellular isolation methods.

(a) viability of cells in the 4 layers and ordinary isolated methods (whole colon) were shown. n=3. Data are shown as means ± SEM. (b) The percentages of cellular components in the 4 layers of colon microenvironment were shown individually. Each bar indicates an independent experiment.

Supplemental Figure 4

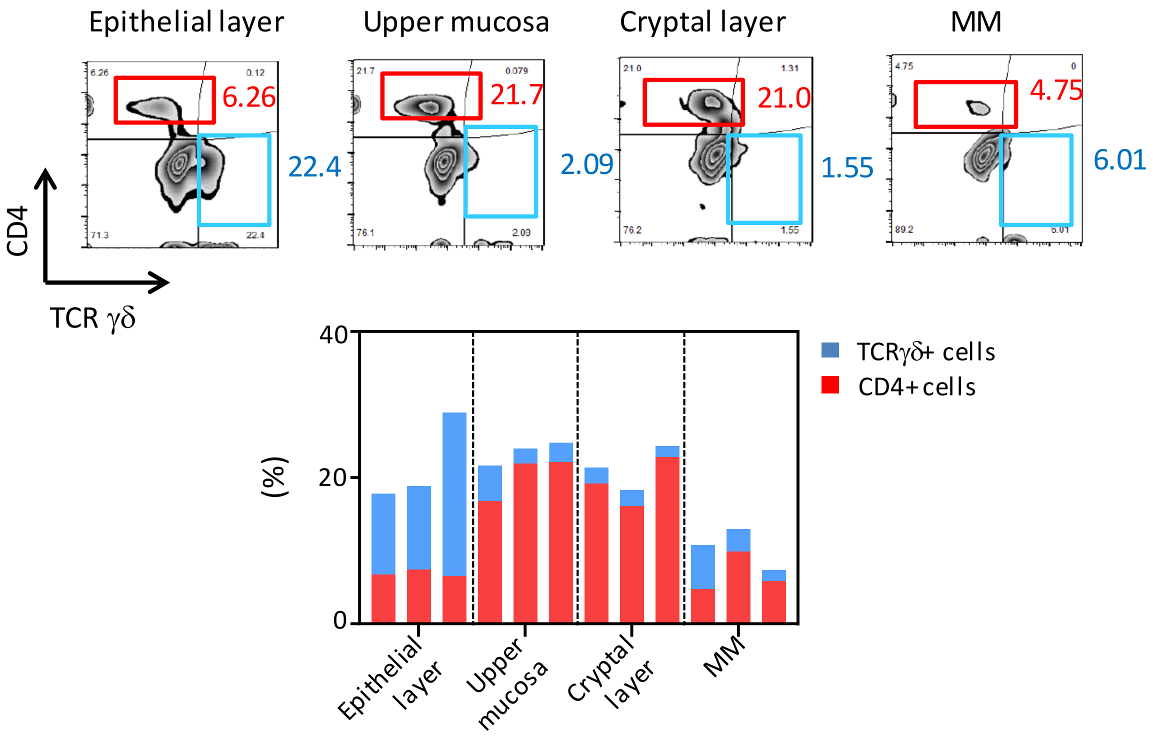

Supplemental Figure 4. Profiling of immunocompetent cells in each stratified layer.

(a) CD4 and TCRγδ^{+} T cells were examined by FACS. The numbers indicate the percentages in each layer. Each bar indicates an independent experiment.

Supplemental Figure 5

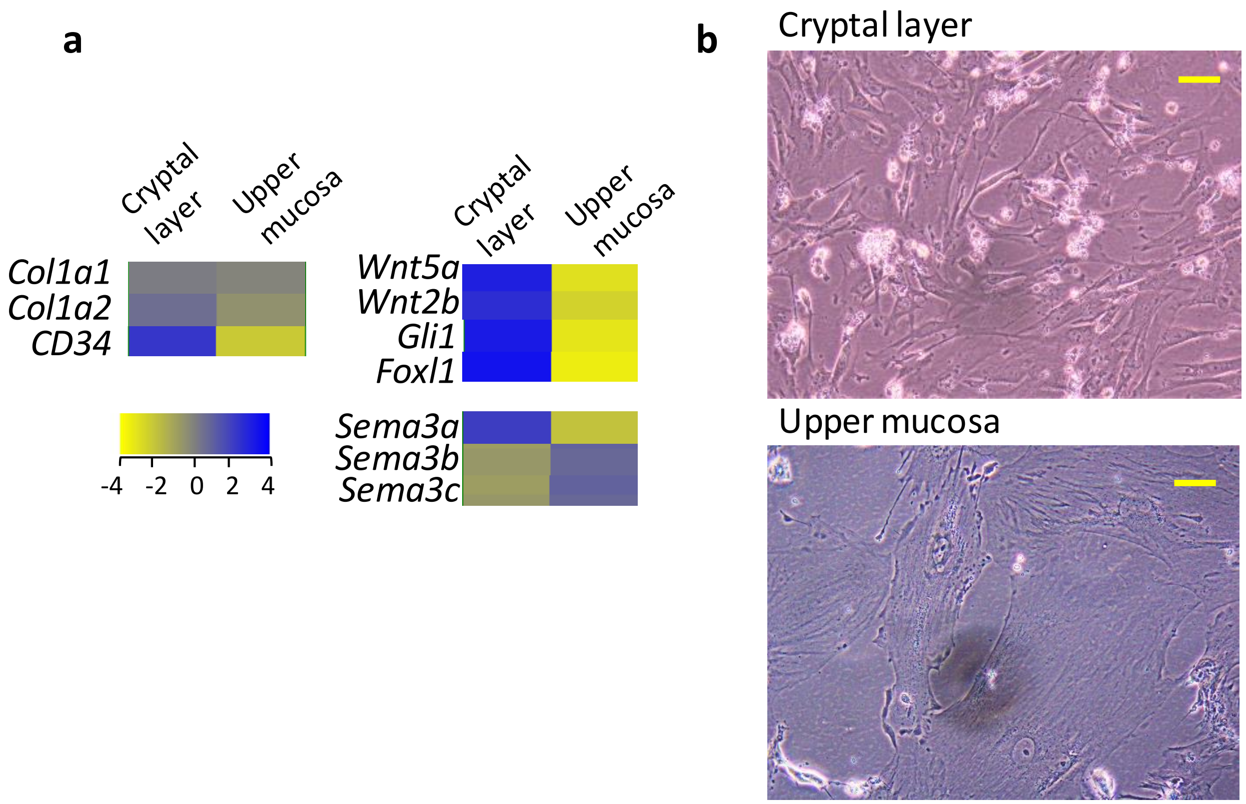

Supplemental Figure 5. Gene profiling of mesenchymal cells in the crypt and upper mucosa.

(a) Col1a1, Col1a2, CD34, and expression of gene sets that are involved in the cryptal niche are shown.
(b) Cultured sorted mesenchymal cells from the cryptal layer and upper mucosa are shown. Bar, 50 μm.

Supplemental Figure 6

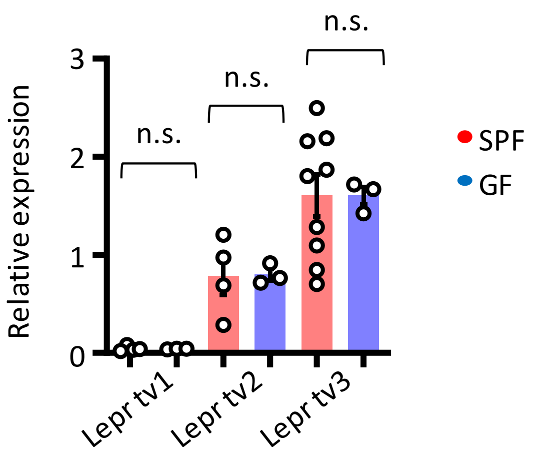

Supplemental Figure 6. Dispensable roles of commensal bacteria in the expression of LepR.

Trans-variants of leptin receptors of SPF C57Bl/6 mice and germ-free (GF) mice are shown. n.s., not significant. Each dot represents an individual subject (n=3-9).

Supplemental Figure 7

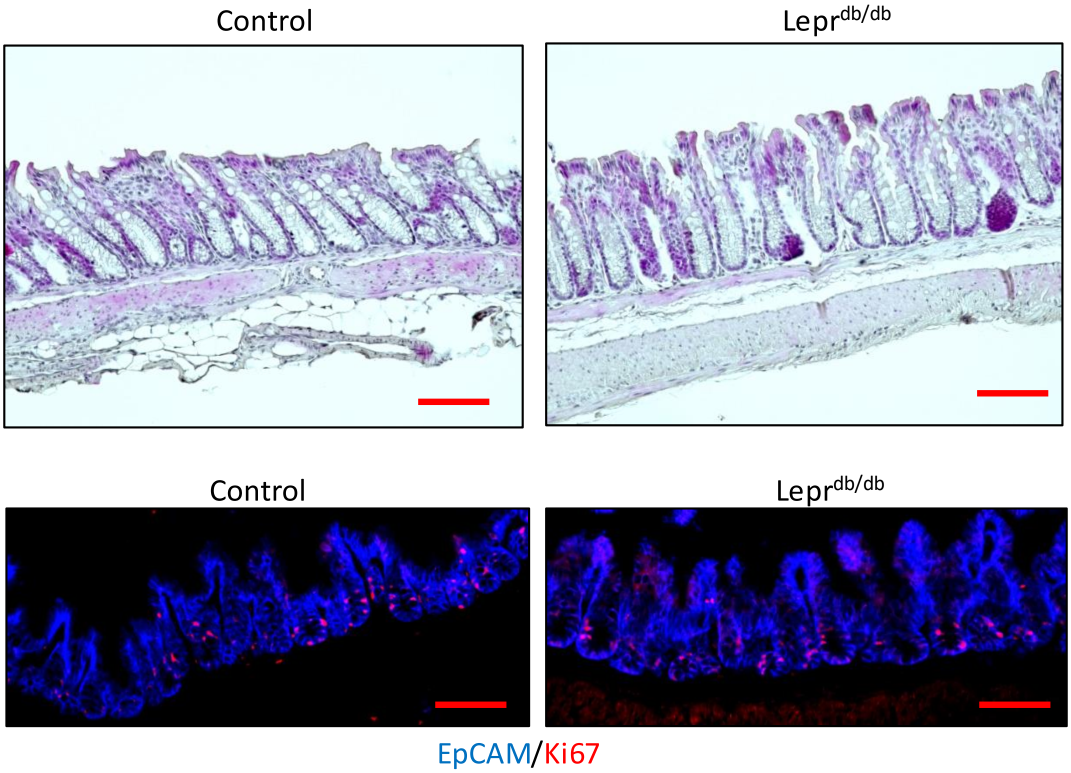

Supplemental Figure 7. Comparable intestinal epithelial cell turnover during steady state in Lepr-deficient mice

H and E staining (upper panel) and Ki67 with EpCAM staining (lower panel) of control and Lepr-deficient (Lepr$^{db/db}$) mice are shown. Representative images of two independent experiments are shown. Scale bar, 100 μm..

Supplemental Figure 8

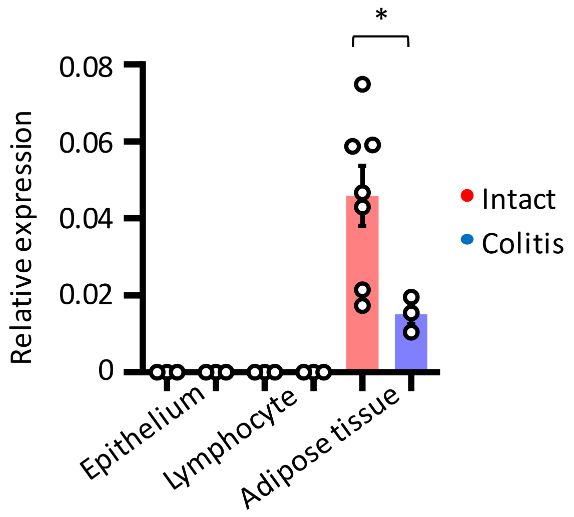

Supplemental Figure 8. Reduction of adipose tissue-derived leptin in colitis

The leptin expression of epithelial and lamina proprial cells (mucosa) and mesenteric adipose tissues was examined through qRT-PCR analysis. Each result was normalized against the expression of *Gapdh.* Each dot represents an individual subject (n=3-7). Data are shown as means ± SEM, $*P < 0.05$.

Supplemental Figure 9

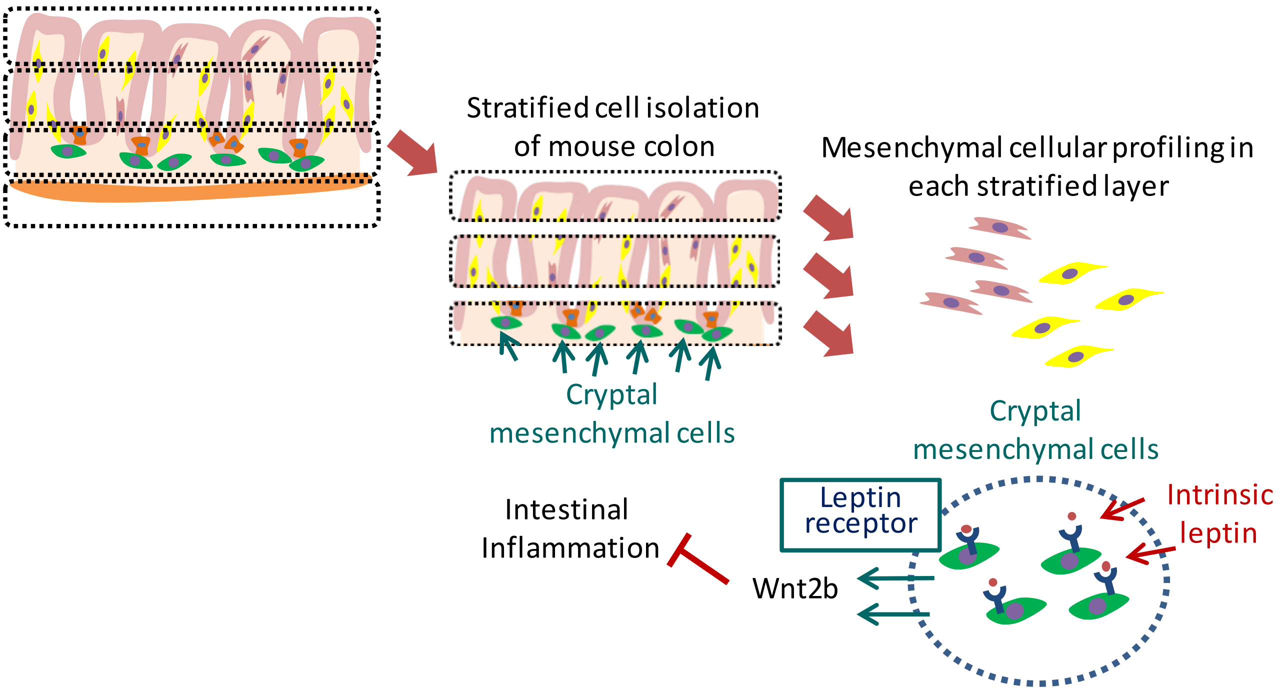

Supplemental Figure 9. Stratified mucosal layer analysis reveals a novel pathway of mucosal homeostasis

To evaluate the function of mesenchymal cells located at the intestinal crypt, we established a novel cell isolation method based on the histological layers in the mouse colon and compared the cell properties between the upper mucosa and crypts. In summary, deficiency of LepR delays epithelial proliferation, thus indicating the importance of intrinsic leptin signaling in the maintenance of mucosal homeostasis.
